# A Systematic Review of Training Methods That May Improve Selective Voluntary Motor Control in Children With Spastic Cerebral Palsy

**DOI:** 10.3389/fneur.2020.572038

**Published:** 2020-12-04

**Authors:** Annina Fahr, Jeffrey W. Keller, Hubertus J. A. van Hedel

**Affiliations:** ^1^Swiss Children's Rehab, University Children's Hospital Zurich, Affoltern am Albis, Switzerland; ^2^Children's Research Center, University Children's Hospital Zurich, Zurich, Switzerland; ^3^Institute for Biomechanics, ETH Zurich, Zurich, Switzerland; ^4^Doctoral Program Clinical Science, Faculty of Medicine, University of Zurich, Zurich, Switzerland

**Keywords:** selective voluntary motor control, involuntary movements, pediatric neurorehabilitation, cerebral palsy, best evidence synthesis

## Abstract

**Background:** Impaired selective voluntary motor control is defined as “the reduced ability to isolate the activation of muscles in response to demands of a voluntary posture or movement.” It is a negative motor sign of an upper motor neuron lesion.

**Objective:** This paper reviews interventions that may improve selective motor control in children and youths with spastic cerebral palsy. The aim was to systematically evaluate the methodological quality and formulate the level of evidence from controlled studies.

**Methods:** Six databases (Scopus, Web of Science, PubMed, Embase, MEDLINE, and CINAHL) were searched with predefined search terms for population, interventions, and outcomes. Two reviewers independently completed study selection and ratings of methodological quality and risk of bias. Evidence was summarized in a best evidence synthesis.

**Results:** Twenty-three studies from initially 2,634 papers were included. The interventions showed a wide variety of approaches, such as constraint-induced movement therapy (CIMT), electrical stimulation, robot-assisted therapy, and functional training. The evidence synthesis revealed conflicting evidence for CIMT, robot-assisted rehabilitation and mirror therapy for the upper extremities in children with cerebral palsy.

**Conclusions:** Final recommendations are difficult due to heterogeneity of the reviewed studies. Studies that include both an intervention and an outcome that specifically focus on selective voluntary motor control are needed to determine the most effective therapy.

## Introduction

A wide variety of acquired or congenital brain injuries can cause lesions of the upper motor neuron, which results in conditions like stroke, traumatic brain injury, or spastic cerebral palsy (CP). Patients with upper motor neuron lesions typically show impairments like decreased muscle force, increased muscle tone/spasticity, and loss of motor control (1, 2). Loss of selective voluntary motor control (SVMC) is defined as “the impaired ability to isolate the activation of muscles in a selected pattern in response to demands of a voluntary posture or movement” (3). This means that patients with impaired SVMC do not show the activation pattern expected in healthy subjects, either due to excessive or lack of muscle activity. SVMC refers to the ability to perform isolated joint movements deliberately and has to be distinguished from habitual selective joint motions during functional tasks such as walking (4). Clinically, reduced SVMC could manifest in mirror movements, which are simultaneous contralateral, involuntary, identical movements that accompany voluntary movements (5) or synergistic muscle activation and movement patterns (obligatory grouped multi-joint movements, e.g., co-activation of M. gastrocnemius and M. quadriceps during knee extension while sitting) (6).

These signs of reduced SVMC can have different neurophysiological origins. Two mechanisms are proposed to contribute to the occurrence of mirror movements (7). First, ipsilateral corticospinal tract projections from the non-lesioned motor cortex to the affected side, and second, insufficient interhemispheric inhibition resulting in bilateral cortical activation. Synergistic and antagonistic co-activations of muscles are addressed to a compensatory reliance on the extrapyramidal rubrospinal tract, which is relatively prominent in infants, in case of corticospinal tract injury (2).

Using the nomenclature of the International Classification of Functioning, Disability and Health (8), SVMC should be considered a body function. Research has shown, though, that impairments in SVMC can contribute to limitations in activities of daily life that some patients experience. For example, children with unilateral spastic CP who show mirror movements require more time for bimanual activities of daily life (9). The abnormal co-activation of shoulder abductor and elbow flexor muscles (“flexion synergy”) reduces the reaching work space in stroke patients (10). A loss of SVMC in the lower extremity has a negative impact on walking ability. For children with spastic CP, impaired SVMC relates to a certain degree to gait abnormalities (11), gait velocity (12), and impaired gross motor function (13).

Despite many cross-sectional studies exploring limitations in SVMC in children with spastic CP, only few interventions actually intend to improve SVMC. These interventions are heterogeneous in terms of the strategies they adapt to enhance SVMC (14–18) and included strength training, performing independent hand movements with suppression or control of mirror movements, and video game-based training of joint movement control. One of the reasons, why not many studies have targeted improving SVMC, could be because many assessments specifically quantifying impairments in SVMC have only recently been established (2).

For children with CP, systematic summaries of the evidence exist about interventions that target improvements in upper limb function, gross motor function, physical activity, or gait speed (19–22). However, a systematic summary of interventions that can lead to improvements in SVMC, which could help to decide which strategy is most promising to train SVMC, is lacking. Other interventional studies might not have primarily focused on improving SVMC, but as they included assessments that quantify SVMC, they could contribute to our understanding what interventions seem beneficial to ameliorate SVMC. Therefore, the aim of this review is to evaluate the quality of the studies and the efficacy of interventions that may improve SVMC. Therefore, the research question is: What is the evidence from controlled interventional trials that may improve SVMC of the upper or lower extremities in children and adolescents with spastic CP?

## Methods

### Search Strategy

We conducted a search in the databases Scopus, Web of Science, PubMed, Embase, MEDLINE (accessed *via* Ovid), and CINAHL, from their inception to present. The initial search was run on December 4, 2018 and updated on November 26, 2019. The search strategy was developed according to the PICOS approach (23). It combined terms for the population of patients with upper motor neuron lesions with terms describing interventions, and keywords and synonyms for SVMC and outcome measures thereof. If applicable, the search strategy also included suitable terms from the databases' controlled vocabulary. The full search strategy for PubMed can be found in the Supplementary Material. This search strategy was adapted for use with other bibliographic databases. We identified additional records by screening the references of relevant reviews retrieved in the search.

### Study Selection

The results retrieved with the search strategy were imported to a reference manager (Mendeley 1.17.12, Mendeley Ltd., London, United Kingdom) where duplicates were removed. Abstract screening and full-text review was conducted with a specialized software (Covidence systematic review software, Veritas Health Innovation, Melbourne, Australia). Two reviewers (AF and JK) independently screened the titles and abstracts to identify studies that were potentially relevant. Cases of disagreement were discussed until consensus was reached. The same two researches then independently reviewed the full-text articles for eligibility. We discussed any disagreements with a third reviewer (HvH).

Studies were included if they met the following criteria: (a) study participants with spastic CP; (b) participants younger than 18 years; (c) include a rehabilitative intervention and the content of the therapy is described; (d) have at least one outcome measure or subscale of an outcome measure (i.e., part of an assessments with a separate score) that assessed SVMC; (e) were peer-reviewed original research articles; and (f) written in English or German. Studies were excluded if (a) the participants had a lesion of the lower motor neuron or degenerative disease; (b) <75% of the study population fulfilled the aforementioned criteria; (c) the intervention was invasive (botulinum toxin therapy, surgery) or concerned drugs; (d) they lacked a control group; and (e) they were only cross-sectional studies.

Since the type of CP was not always reported, we made the following assumptions: (i) unilateral/hemiplegia or di-/quadriplegia refers to the spastic type of CP (24), (ii) reporting other indicators that reflect a spastic component (e.g., including an outcome measure for spasticity). We did not restrict this review on a particular treatment modality, but focused specifically on outcomes for SVMC. Besides established assessments of SVMC, we also included other measures if they covered aspects of SVMC in accordance with the aforementioned definition. We understand measures of SVMC as instruments or subscales thereof that assess selectivity of individual joint movements or voluntary multi-joint movement outside of synergies or mass patterns but not functional gross motor tasks like walking (25). The assessment scoring criteria should consider signs of reduced SVMC like mirror movements and/or compensatory or synergistic movement patterns.

The study protocol was published on Prospero (CRD42019117407). We deviated from the protocol regarding the patient population and the study designs included. First, we initially intended to include studies which had included patients with acquired and congenital upper motor neuron lesions, as this represents the heterogeneous population of children and adolescents with reduced SVMC treated daily in rehabilitation clinics. Later, we limited the search to children with spastic CP, because this allowed more specific conclusions. Second, we initially searched for all study designs, in spite of finally including only controlled studies. Thereby, we aimed to identify whether there are SVMC specific interventions that have not been studied in an RCT yet, since we already expected to find few SVMC specific interventions.

### Data Collection and Quality Assessment

One reviewer (AF) extracted the data into a customized spread sheet, the other reviewer (JK) critically reviewed it. Extracted information included (a) parameters describing the study population (number of participants, age, diagnosis, disease severity); (b) a description of the intervention and control condition (tasks, setting, duration); (c) the outcome measures; and (d) the results.

The studies were assigned a level of evidence based on the study design, as recommended by the guidelines of the American Academy for Cerebral Palsy and Developmental Medicine (AACPDM) (26). We evaluated the methodological quality of studies according to several aspects described in these guidelines. Studies only reaching a weak quality rating (“yes” score on <4 of 7 questions) were excluded from further analyses. A description of the levels of evidence and the methodological conduct questions can be found in the Supplementary Tables 1, 2. Additionally, we evaluated the risk of bias in the seven domains of the risk of bias assessment described by the Cochrane Collaboration (27). Two reviewers (AF and JK) independently conducted the ratings (AACPDM and Cochrane tool) and resolved any discrepancies through discussion.

### Data Analysis

Studies of moderate or strong methodological quality were included in a best evidence synthesis according to van Tulder et al. (28) if they reported results of between-group comparisons. We scored interventions as “improved SVMC” when between-group results were statistically significant and as having “no effect” when results were not statistically significant. When possible, we calculated Hedges' g (29), an unbiased estimate of the standardized mean difference in change, for a rough comparison of significance and effect size for each study.

In accordance with van Tulder et al. (28), the overall level of evidence was considered “strong” if there were consistent findings among multiple high quality (AACPDM quality rating: strong) RCTs, “moderate” for consistent findings among multiple low quality (quality rating: moderate) RCTs and/or one high quality RCT, “limited” if there was one low quality RCT and “conflicting” in case of inconsistent findings. Consistency was defined as ≥75% of studies showing the same effect.

Mixed populations were allocated to specific groups if ≥75% of participants had the same diagnosis, i.e., a study population encompassing eight children with unilateral and one with bilateral spastic CP was labeled as unilateral spastic CP. The studies were further categorized into groups of similar interventions. The categorization into groups of similar types of interventions was based on the intervention that differentiated between the intervention and control group. The overall evidence was determined for specific groups, i.e., based on type of intervention and whether it involved the upper or lower extremity.

## Results

**Figure 1:**
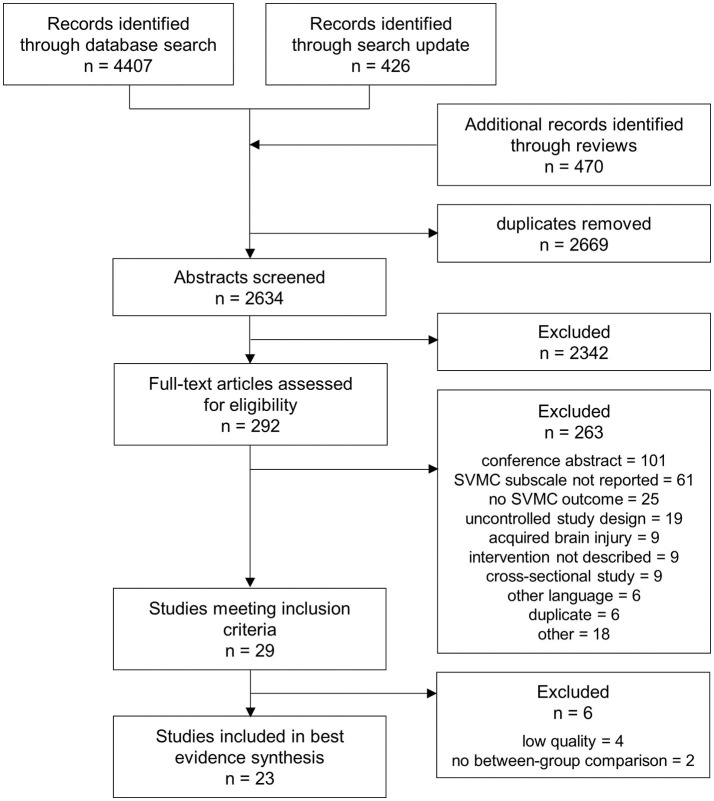
PRISMA (Preferred Reporting Items for Systematic Reviews and Meta-Analyses) flow chart showing the process of study selection.

The search retrieved 4,407 hits. We found another 426 articles when updating the search and 470 additional records were identified from screening the references of 11 relevant reviews contained in the search results. In total, 2,634 abstracts were screened for eligibility after removal of duplicates. After reviewing 292 full-texts, 29 studies were retained. Further, four studies were excluded because of insufficient quality (17, 30–32) and two because between-group comparisons were missing (33, 34). Figure 1 outlines the selection process and shows reasons for exclusion at full-text review.

An overview of all studies is provided in Table 1. One study that matched the inclusion criteria used a single subject research design, namely a randomized controlled N-of-1 trial (AACPDM evidence level I). Among the group design studies, there were 21 RCTs (level II) and one randomized cross-over trial (level II).

**Table 1:**
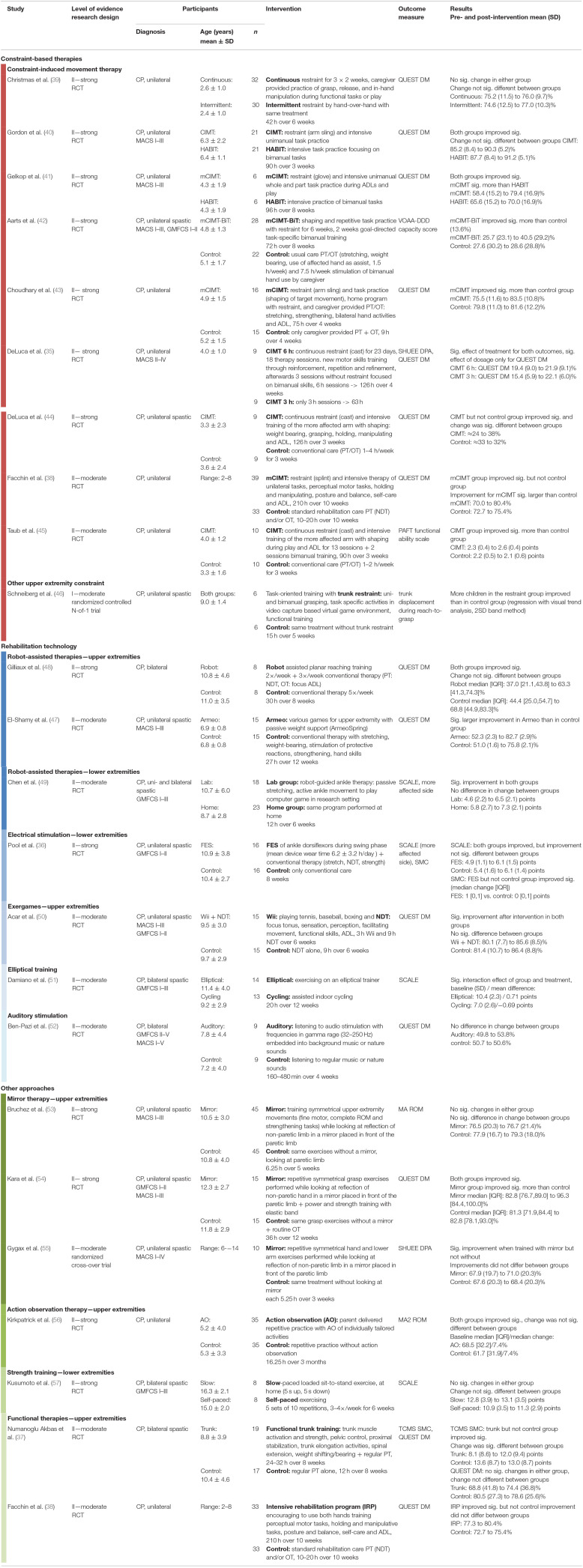
Overview of the included studies.

*ADL, activities of daily living; CP, cerebral palsy; GMFCS, gross motor function classification system; h, hours; MACS, manual ability classification system; RCT, randomized controlled trial; ROM, range of motion; SD, standard deviation; sig, significant*.

***Interventions:** AO, action observation; BiT, bimanual training; (m)CIMT, (modified) constraint-induced movement therapy; FES, functional electrical stimulation; HABIT, hand-arm bimanual intensive therapy; IRP, intensive rehabilitation program; NDT, neurodevelopmental therapy; OT, occupational therapy; PT, physiotherapy*.

***Outcome assessments:** MA(2) ROM, Melbourne assessment (2)—range of motion sub-skill; PAFT, Pediatric Arm Function Test; QUEST DM, quality of upper extremity skills test—dissociated movement domain; SCALE, selective control assessment of the lower extremity; SHUEE DPA, Shriners hospital upper extremity evaluation—dynamic positional analysis; SMC, selective motor control assessment of ankle dorsiflexion; TCMS SMC, trunk control measurement scale—selective motor control section; VOAA-DDD, video-observation Aarts and Aarts module determine developmental disregard—capacity score*.

Characteristics of study participants were heterogeneous in terms of age and severity of disability. The majority of studies included uniquely children with unilateral spastic CP (*n* = 17). Further, there we four studies investigating bilateral spastic CP and mixed groups in two studies.

SVMC was quantified with a wide variety of assessments listed in Table 2. Most studies had a single outcome parameter that measured SVMC; three measured two SVMC outcomes (35–37). The dissociated movement subscale of quality of upper extremity skills test was the most common assessment (used in 13 of 23 studies) followed by the selective control assessment of the lower extremity (used four times). Despite that the quality of upper extremity skills test was not specifically designed to measure SVMC, we considered this subscale appropriate. The dissociated movement section assesses the ability to perform single-joint movements over the whole range of motion while maintaining other joints in a defined position, i.e., abducting the shoulder with the elbow extended.

**Table 2:** Assessments measuring SVMC.

**Assessment**	**Applied in**
Melbourne assessment— sub-skill range of movement	(53, 56)
Pediatric arm function test	(45)
Quality of upper extremity skills test—domain dissociated movement	(35, 37–41, 43, 44, 47, 48, 50, 52, 54)
Selective control assessment of the lower extremity	(36, 49, 51, 57)
Selective motor control assessment of ankle dorsiflexion	(36)
Shriners hospital upper extremity evaluation—dynamic positional analysis	(35, 55)
Trunk control measurement scale—selective movement control	(37)
Trunk displacement during reaching movement	(46)
Video-observation aarts and aarts module determine Developmental disregard—capacity score	(42)

*These assessments measure SVMC on the level of body function. Although children are sometimes observed during activities in these assessments, the test criteria still rate body functions*.

We categorized the interventions into three broad groups: constraint-based therapies, technological interventions, and other approaches. With constraint-based therapies, we mean any use of restraint to limit compensatory strategies, e.g., use of less affected limb. Under rehabilitation technologies, sometimes equaled with robots, we include all electrically powered systems, devices or tools used to meet the needs of rehabilitation. One RCT (38) used a three group design comparing two different interventions to a control group and is thus listed in two categories.

Nine studies (35, 38–45) investigated (modified) constraint-induced movement therapy (CIMT). Constraint was thereby either continuous with a cast or periodically applied during therapy sessions with a splint (or similar). One study used a constraint-based approach other than CIMT. They compared task-oriented upper limb training with and without trunk restraint (46).

Varying rehabilitation technologies were investigated. Three studies evaluated robot-assisted movement therapy of the upper (*n* = 2) or lower extremities (*n* = 1). Assistance was either weight support (47) or physical support of the desired movements (48, 49). Further technological interventions encompassed: (i) electrical stimulation applied peripherally to muscles of the lower extremities while walking (36), (ii) an exergame (50), i.e., game controlled by body movements without any physical support, and therefore, it does not fall into the first group of assisted therapies, (iii) a study that compared indoor cycling to exercising on an elliptical trainer (51), and (iv) one investigating acoustic stimulation (52).

Among the remaining studies, we identified four other approaches. The first two encompass studies on the efficacy of mirror therapy (53–55) and action observation therapy (56). Thirdly, one study investigated lower extremity strength training (57). The fourth category comprises functional training programs (trunk and bimanual training) (37, 38).

Ratings of conduct quality (AACPDM) and risk of bias (Cochrane) of included studies can be found in Tables 3, 4. Methodological conduct quality ranged from strong (7/7 points) to moderate (4/7 points). Studies most often did not report adherence to the intervention and power calculations. Ratings of studies excluded due to insufficient quality (<4 points) can be found in the Supplementary Table 3. Concerning the Cochrane risk of bias assessment, studies received a *low risk* rating on 2/7 to 6/7 domains of possible sources of risk of bias. Most common sources for *high* or *unclear* risk of bias were no or unclear description of the allocation concealment, a lack of information about missing data, and the unavailability of a study protocol to rule out selective reporting.

**Table 3:** Levels of evidence and methodological quality rating of included studies in accordance with AACPDM guidelines.

**Group research design studies**	**Evidence level**	**Quality**	**Methodological conduct question**						
			**1**	**2**	**3**	**4**	**5**	**6**	**7**
Christmas et al. (39)	II	Strong−7/7	Yes	Yes	Yes	Yes	Yes	Yes	Yes
Gordon et al. (40)	II	Strong−7/7	Yes	Yes	Yes	Yes	Yes	Yes	Yes
Gelkop et al. (41)	II	Strong−6/7	Yes	Yes	Yes	Yes	No	Yes	Yes
Aarts et al. (42)	II	Strong−6/7	Yes	Yes	Yes	Yes	No	Yes	Yes
Choudhary et al. (43)	II	Strong−6/7	Yes	No	Yes	Yes	Yes	Yes	Yes
DeLuca et al. (35)	II	Strong−6/7	Yes	Yes	Yes	Yes	No	Yes	Yes
DeLuca et al. (44)	II	Strong−6/7	Yes	Yes	Yes	Yes	No	Yes	Yes
Facchin et al. (38)	II	Moderate−5/7	Yes	No	Yes	Yes	No	Yes	Yes
Taub et al. (45)	II	Moderate−4/7	Yes	No	Yes	Yes	No	Yes	No
Gilliaux et al. (48)	II	Strong−6/7	Yes	Yes	Yes	Yes	No	Yes	Yes
El-Shamy et al. (47)	II	Moderate−5/7	Yes	No	Yes	Yes	No	Yes	Yes
Chen et al. (49)	II	Moderate−4/7	Yes	Yes	Yes	No	No	No	Yes
Pool et al. (36)	II	Strong−6/7	Yes	Yes	Yes	No	Yes	Yes	Yes
Acar et al. (50)	II	Moderate−4/7	Yes	No	Yes	No	No	Yes	Yes
Damiano et al. (51)	II	Moderate−5/7	Yes	Yes	Yes	No	Yes	Yes	No
Ben-Pazi et al. (52)	II	Moderate−4/7	Yes	No	Yes	Yes	No	Yes	No
Bruchez et al. (53)	II	Strong−7/7	Yes	Yes	Yes	Yes	Yes	Yes	Yes
Kara et al. (54)	II	Strong−7/7	Yes	Yes	Yes	Yes	Yes	Yes	Yes
Gygax et al. (55)	II	Moderate−5/7	Yes	No	Yes	Yes	No	Yes	Yes
Kirkpatrick et al. (56)	II	Strong−7/7	Yes	Yes	Yes	Yes	Yes	Yes	Yes
Kusumoto et al. (57)	II	Strong−6/7	Yes	Yes	Yes	Yes	No	Yes	Yes
Numanoglu Akbas et al. (37)	II	Moderate−5/7	Yes	No	Yes	Yes	No	Yes	Yes
**Single subject research design studies**	**Evidence level**	**Quality**	**Methodological conduct question**						
Schneiberg et al. (46)	I	Moderate−7/14	**1**	**2**	**3**	**4**	**5**	**6**	**7**
			No	Yes	Yes	Yes	No/No	Yes	No
			**8**	**9**	**10**	**11**	**12**	**13**	**14**
			No/No	No	Yes	Yes	No	Yes	No

*The levels of evidence represent the ability of the study design to establish causality between the intervention and the outcome from I—most definitive to V—only suggesting the possibility. AACPDM, American Academy for Cerebral Palsy and Developmental Medicine*.

*The color code represents the groups of similar types of interventions. Red colours stand for constraint-based therapies, blue colors for rehabilitation technologies and green colors for other approaches*.

**Table 4:** Cochrane risk of bias assessment of included studies.

**Study**	**Domain**						
	**Random sequence generation**	**Allocation concealment**	**Blinding of participants and personnel**	**Blinding of outcome assessment**	**Incomplete outcome data**	**Selective reporting**	**Other bias**
Christmas et al. (39)	Low risk	High risk	Low risk	Low risk	Unclear	Low risk	Low risk
Gordon et al. (40)	Low risk	Low risk	Low risk	Low risk	Unclear	Unclear	Low risk
Gelkop et al. (41)	Low risk	Low risk	Low risk	Low risk	High risk	Unclear	Low risk
Aarts et al. (42)	Low risk	Low risk	Low risk	Low risk	Unclear	Unclear	Low risk
Choudhary et al. (43)	Low risk	High risk	High risk	Low risk	Low risk	High risk	Low risk
DeLuca et al. (35)	Unclear	Unclear	Low risk	Low risk	Low risk	Unclear	Low risk
DeLuca et al. (44)	Unclear	Unclear	Low risk	Low risk	Unclear	Unclear	Low risk
Facchin et al. (38)	Unclear	Unclear	Low risk	Low risk	Unclear	Low risk	High risk
Taub et al. (45)	Unclear	High risk	Low risk	Low risk	Unclear	Unclear	High risk
Schneiberg et al. (46)	Unclear	Low risk	Low risk	High risk	Unclear	Unclear	Low risk
Gilliaux et al. (48)	Low risk	Low risk	Low risk	Low risk	Unclear	Low risk	Low risk
El-Shamy et al. (47)	Low risk	Unclear	Low risk	Low risk	Unclear	Unclear	Low risk
Chen et al. (49)	Low risk	Unclear	Low risk	High risk	Unclear	Low risk	High risk
Pool et al. (36)	Low risk	Low risk	Low risk	High risk	Low risk	Low risk	Low risk
Acar et al. (50)	Unclear	Unclear	Low risk	Unclear	Unclear	Unclear	Low risk
Damiano et al. (51)	Unclear	High risk	Low risk	High risk	Low risk	High risk	High risk
Ben-Pazi et al. (52)	Low risk	High risk	Low risk	Low risk	Low risk	High risk	High risk
Bruchez et al. (53)	Low risk	High risk	Low risk	Low risk	Low risk	Low risk	Low risk
Kara et al. (54)	Low risk	Low risk	Low risk	Low risk	Unclear	High risk	Low risk
Gygax et al. (55)	Unclear	Unclear	Low risk	Low risk	Unclear	Unclear	Low risk
Kirkpatrick et al. (56)	Low risk	Low risk	Low risk	Low risk	Low risk	High risk	Low risk
Kusumoto et al. (57)	Low risk	Unclear	Low risk	Low risk	Unclear	Unclear	Low risk
Numanoglu Akbas et al. (37)	Low risk	Low risk	Low risk	Low risk	Unclear	High risk	Low risk

*Detailed explanation of the seven domains of potential sources of bias are described in the risk of bias assessment of the Cochrane Collaboration (27)*.

*The color code represents the groups of similar types of interventions. Red colours stand for constraint-based therapies, blue colors for rehabilitation technologies and green colors for other approaches*.

### Best Evidence Synthesis

The overall level of evidence is summarized below and Table 5 shows the best evidence synthesis. When available, the effect sizes confirmed the results based on statistical significance. Hedges' g exceeded 0.79 for significant comparisons and was smaller than 0.50 for all non-significant comparisons.

**Table 5:**
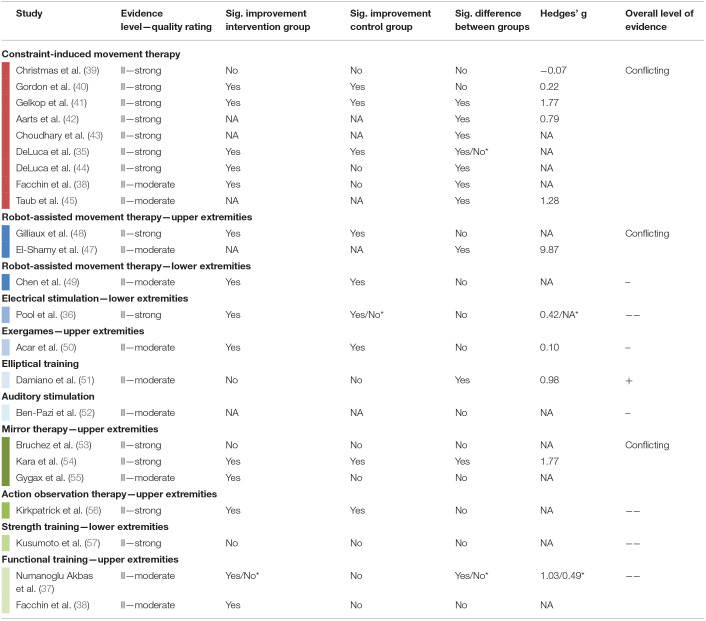
Summary of evidence and best evidence synthesis according to van Tulder et al. (28) of controlled group design studies with moderate or strong methodological quality.

– no effect, + positive effect, *two outcomes, NA, information not available.

*Overall level of evidence from best evidence synthesis (28): + + +/− − − = strong: consistent findings among multiple high quality (quality rating: strong) RCTs, ++/−− = moderate: consistent findings among multiple low quality (quality rating: moderate) RCTs and/or one high quality RCT, +/− = limited: one low quality RCT and conflicting in case of inconsistent findings*.

*The color code represents the groups of similar types of interventions. Red colours stand for constraint-based therapies, blue colors for rehabilitation technologies and green colors for other approaches*.

#### Constraint-Based Therapies

There were nine RCTs (35, 38–45) summarized in the evidence synthesis for CIMT in children with unilateral CP. The trials included between 12 and 72 children (total *n* = 325), aged from 0.6 to 10 years, with MACS levels I to IV. Intensity of therapy varied between 42 and 210 h administered over 3 to 10 weeks. We observed conflicting evidence concerning the efficacy of CIMT on SVMC, mostly assessed with the quality of upper extremity skills test. Some studies showed a significantly improved SVMC outcome after CIMT compared to the control condition, while other studies did not find differences between the interventions.

Regarding other constraint-based approaches, a randomized N-of-1 trial found that more children reduced their trunk compensation during reach-to-grasp movements after task-oriented training with trunk restraint compared to children training without trunk constraint (46).

#### Rehabilitation Technology

Two RCTs (total *n* = 46 children) compared robot-assisted upper limb training to conventional therapy, one in children with unilateral CP (47), the other in children with bilateral CP (48). Due to inconsistent findings, the overall evidence for robot-assisted upper limb training is conflicting. For the lower extremities, only one RCT was eligible for the best evidence synthesis (49). In 41 children with varying types of CP, the efficacy of a robotic ankle training program was compared between different settings. Both the home-based and the lab-based group significantly improved movement selectivity but no difference between settings could be shown.

From the other interventions, a study in 32 children with unilateral CP indicated that using a device for electrical stimulation of ankle dorsiflexion during the swing phase of gait was not more efficacious than conventional care (36). Concerning exergames, the single RCT found that playing games with the Nintendo Wii console had no additional effect on SVMC compared to neurodevelopmental treatment alone in 30 children with unilateral CP (50). Acoustic stimulation did also not change upper limb selective control (52). The change of lower limb SVMC in 27 children with bilateral CP after training on an elliptical compared to indoor cycling was significantly different, favoring the elliptical trainer (51).

#### Other Approaches

Two RCTs (53, 54) and one cross-over trial (55) investigated the efficacy of mirror therapy in totally 130 children with unilateral spastic CP (MACS I–IV). The evidence is conflicting whether offering a visual impression of a functional limb (by illusion through the mirror) has a superior effect on SVMC compared to repetitively practicing the same movement without vision of correct movement execution. The efficacy of action observation therapy, hence preceding movement practice by watching somebody performing that movement, was not different from movement practice alone (56).

The strength training category included only one RCT eligible for the evidence synthesis. Kusumoto et al. (57) compared loaded sit-to-stand exercises performed at slow vs. self-paced speed in 16 children with bilateral spastic CP (GMFCS I–III). From the results, no speed of movement execution could be favored in terms of its effect on SVMC.

Functional training programs were studied in two RCTs but with a different focus. The first study compared the efficacy of trunk training to regular physiotherapy alone in 36 children with bilateral spastic CP (37). Trunk training led to significant improvements in selective trunk control but not the scores of the quality of upper extremity skills test. The second study emphasized the use of both hands in 66 young children with unilateral CP (38). This intensive bimanual training (210 h) did not differ from a less intensive conventional rehabilitation program (10–20 h) concerning its effect on SVMC.

## Discussion

This review provides an overview of interventions that might affect SVMC in children with spastic CP and systematically summarizes the evidence on the efficacy of these interventions to improve SVMC. Most of the 23 studies recruited children with unilateral CP. The methodological quality of the studies varied widely, as did the characteristics of participants and dosage of treatments. The interventions covered several therapeutic approaches, broadly grouped into constraint-based interventions, rehabilitation technologies, and other approaches. The consecutive evidence synthesis was performed for specific subgroups, i.e., based on interventions and upper or lower extremity. The overall level of evidence for interventions to improve SVMC in children with spastic CP is conflicting for the subgroups CIMT, robot-assisted therapy approaches and mirror therapy for the upper extremities and absent for trunk or bimanual training. Only a single study in each subgroup prevented a synthesis of results across studies for the effect of robot-assisted lower extremity training, electrical stimulation, exergames, auditory stimulation, elliptical training, action observation therapy, or strength training.

Often, the aims of the studies were formulated rather general like improving *upper limb-* (39) or *motor function* (37). Sometimes, the studies investigated a (novel) intervention without explicitly stating the target of the therapy (48, 53, 54). We still included these studies not specifically targeting selective control because they assessed outcomes that cover SVMC and thus could contribute to identify interventions that might improve SVMC. However, the unspecific nature of activity-based interventions can explain why we found only few indications for SVMC improvements.

Among the outcome measures that were included in the studies, there are only a few assessments that specifically measure SVMC (i.e., the selective control assessment of the lower extremity, the selective motor control assessment of ankle dorsiflexion, and the trunk control measurement scale). Indeed, the assessment of SVMC has only recently advanced and there exists only a limited number of tools (25). For the upper extremities, the majority of assessments covered a broad range of motor functions, which included aspects of SVMC in their scoring. A limitation of the SVMC measures for the lower extremity is that their responsiveness to changes has not been evaluated yet (25). In line with the lack of information of the responsiveness, also the minimal clinically important difference has not been determined for most assessments (especially subscales). However, both aspects are relevant when evaluating treatment effects, as done in this review.

### Constraint-Based Therapies

CIMT is a commonly used approach for improving upper limb function in children with hemiplegic CP with a lot of variation in its implementation (setting, duration of restraint and therapy) (58). The effect of CIMT on arm function has repeatedly been systematically reviewed in children with CP (59–61). The conclusion was that CIMT provides a beneficial effect compared to a low-dose comparison but is not (59, 60) or only slightly more effective (61) than a control intervention of the same dose. There is a similar trend for the effect of CIMT on SVMC. Studies that found no differences in SVMC between intervention and control groups also applied intensive, dose-matched protocols (39, 40). On the contrary, those studies with a low-dose comparison comprising regular therapy found CIMT to be more efficacious in improving SVMC (38, 43–45). Two studies are inconsistent with this trend by showing CIMT to be more effective than dose-matched control treatments. Although the interventions of Aarts et al.'s study (42) were dose-matched, CIMT was provided by dedicated therapists while the control intervention was mostly parental stimulation of bimanual activities, which might be less intensive. The result of Gelkop et al. (41) might be explained by differences in the outcome existing previous to the intervention that influenced the potential for improvement.

Several studies did not consist of only CIMT sessions, but they either included a small number of bimanual sessions after completion of the CIMT program (35, 42, 45) or low-dose concomitant conventional therapy including bimanual activities (43). The idea of this combination is that bimanual training promotes incorporation of skills acquired during CIMT to meaningful activities (62). Further studies are needed to determine whether combining CIMT and bimanual training is more efficacious for improving SVMC than each intervention alone.

### Rehabilitation Technology

Technological advances have led to an increased use of technology for rehabilitation over the last years. The field being under development might explain why our initial search retrieved many uncontrolled studies. These investigations focused on technological development and provided a proof of concept rather than establishing efficacy. Studies including rehabilitation technologies showed a wide variety of approaches. Only for robot-assisted upper limb training, there was more than one study to summarize. In Gilliaux et al.'s study (48), 40% of the training sessions differed between the groups (i.e., the intervention group received robotic therapy for 2/5 weekly sessions) while 60% were similar (i.e., 3/5 weekly sessions for the intervention and 5/5 weekly sessions for the control group consisted of conventional therapy), i.e., a relatively low contrast between intervention and control group. Improvements in SVMC were not significantly different. On the contrary, El-Shamy (47) found a significantly higher improvement in the intervention group, which received only robotic therapy, compared to the control group, which received only conventional therapy, i.e., a large contrast between groups. Although both studies had dose-matched intervention and control conditions, conflicting evidence might be the result of a different degree of contrast between the control and intervention group. Selection of the control condition is also an issue for robot-assisted training of the lower extremities. Comparing the efficacy of a robotic ankle movement training between a home-based and a research setting did not show differences in SVMC improvements but does not allow to draw conclusions about its effect on SVMC compared to standard therapies (49).

### Other Approaches

The evidence synthesis did not reveal positive effects on SVMC for any other intervention category. Among three studies on mirror therapy, only Kara et al. (54) found an effect of the intervention on SVMC. Their intervention lasted considerably longer than in the other studies, but intervention and control also differed in a second aspect besides mirror therapy. They complemented mirror therapy with power and strength exercises, while the control group additionally received routine occupational therapy. This design does not allow differentiating which part of the intervention caused the effect. For upper limb action observation and the other two studies on mirror therapy, the reason why they were not more efficacious than the control might be that conditions were too similar (53, 55, 56). Apart from letting the child observe the visual illusion, the intervention did not differ from the control condition. Observation had to be guaranteed by the parents delivering the therapy.

Previous studies showed strength being related to the selective control assessment of the lower extremity (63, 64). Therefore, it could be expected that strengthening programs for the lower extremity might also improve selective control. The efficacy of sit-to-stand exercises to compared to another intervention remains unknown, since Kusumoto et al. (57) only compared different movement speeds. As they also could not find significant within-group improvements, sit-to-stand exercises do not seem to improve SVMC.

Functional training approaches focused on the trunk and bimanual activities. Trunk control is important for upper limb movements by providing a stable base. In children with CP, better trunk control was related to better upper extremity function (65, 66). Therefore, functional trunk training might improve both, selective control of the trunk and the upper extremities, which was however not proven for the latter (37). Concerning bimanual training, the non-significant result of Facchin et al.'s study (38) is surprising because of the huge dose difference between intervention and control group.

### Methodological Considerations and Limitations

As the definition of SVMC includes various aspects, defining appropriate search terms was difficult. Our comprehensive search strategy included a variety of terms that describe SVMC. Thereby, we aimed to find publications that investigated selective control but used another wording. However, we still might have missed some studies, which could change the evidence. We grouped the studies by type of intervention, but there are more factors that should be taken into account: a) dosage, which ranged from below 10 h to more than 200 h of therapy; b) selection of the control condition and whether it is dose-matched or a different dosage; c) form of CP and severity of impairment; and d) the outcome measure used. The outcome measure was indeed an important criterion to decide whether a study would be in- or excluded from this systematic review. We studied the test manuals and discussed various assessments extensively before deciding whether an assessment was considered to measure SVMC. We acknowledge that many more studies have been conducted about each type of intervention but these were not included because they lacked a measure of SVMC, which was the focus of this review.

One limitation is the small number of studies that included an intervention specifically targeting SVMC. Gordon (67) claimed that reduced SVMC is one of the least appreciated clinical features of CP, which could explain why we found only the robotic ankle movement training program (49) as an example of an intervention specifically targeting SVMC. The application of more specific outcome measures and interventions would allow more precise conclusions about the efficacy of interventions to enhance SVMC. Initially, we found two other studies on SVMC specific approaches, but these had to be excluded during the review process. The first one encompassed a commercial video game, which is controlled by surface electromyography signals to reinforce desired muscle activity and reduce co-contraction of an agonist-antagonist muscle pair, thus to train selective muscle activation (15). The second study by Adler et al. (18) aimed to reduce mirror movements with a bimanual therapy program developed for this purpose. Since, tailored interventions likely have the potential for yielding larger improvements, they should be further investigated in controlled studies.

Future studies should also clarify whether children can translate improvements in SVMC to more independence in daily life activities, which is an important goal for the patients and their families.

## Conclusions

We systematically reviewed the evidence on the efficacy of interventions that may improve SVMC and found a wide variety of approaches. Some studies could show a positive effect on SVMC but the overall level of evidence was conflicting for (m)CIMT, robot-assisted therapy and mirror therapy of the upper extremities in children with CP. We noted a large variability in outcome measures, dosage, and selection of the control intervention.

## Data Availability Statement

The original contributions presented in the study are included in the article/Supplementary Materials, further inquiries can be directed to the corresponding author/s.

## Author Contributions

AF developed the search strategy, performed the search, study selection and data extraction, and wrote the manuscript. JK was involved in developing the search strategy, study selection, and data extraction. HvH was involved in the search design, study selection, interpretation of results, supervision, and provided the funding. All authors critically reviewed the manuscript and agreed with the final version.

## Conflict of Interest

The authors declare that the research was conducted in the absence of any commercial or financial relationships that could be construed as a potential conflict of interest.
