# Supplementary material for: A Systematic Review of Training Methods That May Improve Selective Voluntary Motor Control in Children With Spastic Cerebral Palsy
Source: Front Neurol. 2020 Dec 4;11:572038. doi: 10.3389/fneur.2020.572038 (PMC7746811; doi:10.3389/fneur.2020.572038)
Supplement: Supplementary file 2 [file Table_2.DOCX]

Supplementary Material

# Search Strategy PubMed

(("upper" AND "motor" AND "neuron" AND "lesion") OR "Brain Injuries, Traumatic"[MeSH] OR "Brain Hemorrhage, Traumatic"[MeSH] OR "Brain Injury, Chronic"[MeSH] OR "traumatic brain injury" OR "chronic brain injury" OR "acquired brain injury" OR “congenital brain injury” OR “congenital brain lesion” OR "stroke"[MeSH] OR "stroke" OR "spinal cord injuries"[MeSH] OR "spinal cord injury" OR "cerebral palsy"[MeSH] OR "cerebral palsy" OR "brain neoplasms"[MeSH] OR "brain neoplasm" OR "brain tumor") AND ("Rehabilitation"[Mesh:NoExp] OR "Neurological Rehabilitation"[Mesh] OR "rehabilitation" OR "train" OR “training” OR "therapy" OR "treat" OR "treatment" OR "physiotherapy" OR "physical therapy" OR "Occupational Therapy"[MeSH] OR "occupational therapy" OR "exercise" OR "intervention" OR "restore" OR "Convalescence"[Mesh] OR “recovery” OR “restoration” OR "Randomized Controlled Trials as Topic"[Mesh:NoExp] OR "Controlled Clinical Trials as Topic"[Mesh:NoExp] OR “controlled trial” OR “functional electrical stimulation”) AND ("selective movement" OR "selective control" OR "selective muscle activation" OR “selective voluntary motor control” OR "selective motor control" OR "selective muscle control" OR "involuntary movement" OR "isolated movement" OR "motor compensation" OR "compensatory movement" OR "muscle coactivation" OR "muscle co-activation" OR "muscular co-activation" OR "muscular coactivation" OR “muscle cocontraction” OR “muscle co-contraction” OR “muscular cocontraction” OR “muscular co-contraction” OR "mirror movement" OR "muscle synergy" OR "muscular synergy" OR "movement synergy" OR "flexion synergy" OR "extension synergy" OR "muscle coupling" OR "muscular coupling" OR "force modulation" OR "torque steadiness" OR ((“kinematic” OR “kinematics”) AND “selective”) OR "Selective Motor Control test" OR "SMC test" OR "modified Trost test" OR "mTrost" OR "Gillette's Selective motor control test" OR "Gillette's SMC test" OR "Selective Control Assessment of the Lower Extremity" OR "selective control of the upper extremity test" OR "SCUES" OR "test of arm selective control" OR "TASC" OR "Quality of upper extremity skills test" OR "Melbourne Assessment" OR "MUUL" OR "MA2" OR "Shriners Hospital Upper Extremity Evaluation" OR "SHUEE" OR (“woods” AND “teuber”) OR “voluntary response index” OR “similarity index”)

# Supplementary Tables

## Table S1: AACPDM Levels of Evidence

| **Level** | **Group research design studies** |
| --- | --- |
| I | Systematic review of RCTs  Large RCT (with narrow confidence intervals) (n >100) |
| II | Smaller RCT’s (with wider confidence intervals) (n<100)  Systematic reviews of cohort studies  “Outcomes research” (very large ecologic studies) |
| III | Cohort studies (must have concurrent control group)  Systematic reviews of case control studies |
| IV | Case series  Cohort study without concurrent control group (e.g. with historical control group)  Case-control Study |
| V | Expert Opinion  Case study or report  Bench research  Expert opinion based on theory or physiologic research  Common sense/anecdotes |
|  | **Single subject design studies** |
| I | Randomized controlled N-of-1, alternating treatment design, and concurrent or non-concurrent multiple baseline design  Generalizability if the alternating treatment design is replicated across three or more subjects and the multiple baseline design consists of a minimum of three subjects, behaviors, or settings  These designs can provide causal inferences. |
| II | Non-randomized, controlled, concurrent multiple baseline design  Generalizability if design consists of a minimum of three subjects, behaviors, or settings.  Limited causal inferences. |
| III | Non-randomized, non-concurrent, controlled multiple baseline design  Generalizability if design consists of a minimum of three subjects, behaviors or settings.  Limited causal inferences. |
| IV | Non-randomized, controlled SSRDs with at least three phases (ABA, ABAB, BAB, etc.).  Generalizability if replicated across three or more different subjects.  Only hints at causal inferences. |
| V | Non-randomized controlled AB SSRD  Generalizability if replicated across three or more different subjects.  Suggests causal inferences allowing for testing of ideas. |

*Abbreviations: AACPDM: American Academy for Cerebral Palsy and Developmental Medicine, RCT: randomized controlled trial, SSRD: single subject research design.*

## Table S2: AACPDM Methodological Quality Rating: Conduct Questions

| **Group research design studies** |
| --- |
| 1. Were inclusion and exclusion criteria of the study population well described and followed? 2. Was the intervention well described and was there adherence to the intervention assignment?* (for 2-group designs, was the control exposure also well described?) 3. Were the measures used clearly described, valid and reliable for measuring the outcomes of interest? 4. Was the outcome assessor unaware of the intervention status of the participants (i.e., were the assessors masked)? 5. Did the authors conduct and report appropriate statistical evaluation including power calculations?*** 6. Were dropout/loss to follow-up reported and less than 20%? For 2-group designs, was dropout balanced? 7. Considering the potential within the study design, were appropriate methods for controlling confounding variables and limiting potential biases used?   The conduct of an individual study is judged as strong (‘yes’ score on 6 or 7 of the questions), moderate (‘yes’ score on 4 or 5) or weak (‘yes’ score on less than 4). |
| **Single subject design studies** |
| 1. Was/were the participant(s) sufficiently well described to allow comparison with other studies or with the reader’s own patient population? 2. Were the independent variables operationally defined to allow replication? 3. Were intervention conditions operationally defined to allow replication? 4. Were the dependent variables operationally defined as dependent measures? 5. 5. Was inter-rater or intra-rater reliability of the dependent measures assessed before and during each phase of the study?*3 6. Was the outcome assessor unaware of the phase of the study (intervention vs. control) in which the participant was involved? 7. Was stability of the data demonstrated in baseline, namely lack of variability or a trend opposite to the direction one would expect after application of the intervention? 8. 8. Was the type of SSRD clearly and correctly stated, for example, A-B, multiple baseline across subjects?** 9. Were there an adequate number of data points in each phase (minimum of five) for each participant? 10. Were the effects of the intervention replicated across three or more subjects? 11. Did the authors conduct and report appropriate visual analysis, for example, level, trend and variability? 12. Did the graphs used for visual analysis follow standard conventions, for example x- and y- axes labeled clearly and logically, phases clearly labeled (A,B, etc.) and delineated with vertical lines, data paths separated between phases, consistency of scales? 13. Did the authors report tests of statistical analysis, for example celeration line approach, two-standard deviation band method, C-statistic, or other? 14. Were all criteria met for the statistical analyses used?   *The conduct of an individual study is judged as strong (‘yes’ score on 11 to 14 of the questions), moderate (‘yes’ score on 7 to 10) or weak (‘yes’ score on less than 7)* |

** Both parts of the question need to be met to score ‘yes’, **0.5 points are assigned to each part (26). Abbreviations: AACPDM: American Academy for Cerebral Palsy and Developmental Medicine.*

# Table S3: AACPDM methodological quality rating of studies excluded based their low quality.

| **Group research design studies** | **Evidence Level** | **Quality** | **Methodological conduct question** | | | | | | |
| --- | --- | --- | --- | --- | --- | --- | --- | --- | --- |
|  |  |  | **1** | **2** | **3** | **4** | **5** | **6** | **7** |
| Kwon et al., 2014 (30) | III | weak - 3/7 | yes | no | yes | yes | no | no | no |
| Motta et al., 2010 (31) | III | weak - 2/7 | yes | no | yes | no | no | no | no |
| Kumari et al., 2014 (17) | II | weak - 3/7 | yes | no | yes | no | no | yes | no |
| Hughes et al., 2017 (32) | II | weak - 2/7 | yes | no | yes | no | no | no | no |

*Abbreviations: AACPDM: American Academy for Cerebral Palsy and Developmental Medicine.*
